# Mapping load transfer from the plantar surface of the foot to the wall of the total contact cast (TCC)

**DOI:** 10.1186/1757-1146-4-S1-O6

**Published:** 2011-05-20

**Authors:** Lindy Begg, Patrick McLaughlin, Leon Manning, Axel Kalpen, John Fletcher, Joshua Burns

**Affiliations:** 1 Foot Wound Clinic, Department of Surgery, Westmead Hospital, 2145, Australia; 2 School of Biomedical and Health Sciences, Faculty of Health, Engineering and Science, Victoria University, Melbourne 8001, Australia; 3 Novel Biomechanics Lab, Munich, Germany; 4 Institute for Neuroscience and Muscle Research, The Children’s Hospital at Westmead/Paediatric Gait Analysis Service of New South Wales/Faculty of Health Sciences, The University of Sydney, NSW, 2145, Australia

## Background

The success of the total contact cast (TCC) is attributed to its ability to reduce pressure at the ulcer site coupled with forced patient compliance. The mechanism of offloading plantar pressure with a TCC is purported to be by redistributing weight-bearing force across the entire plantar surface of the foot and increasing the plantar surface contact area. An examination of the contact area data and regional pressure patterns between conditions in previous work suggested that this is not entirely the case. These results generally support the theory that total contact casting transfers load to the cast walls; however, this has not previously been measured.

## Methods

Two 15x3cm strips of capacitance sensors (novel pliance, Germany) with a resolution of 1 sensor/cm^2^ were placed at various locations around the lower leg to map the load between the leg and the cast wall in one healthy and one diabetic participant. Seven different locations on the lower leg were required. Plantar pressure data were collected simultaneously using an in-shoe pressure sensor (novel pedar, Germany) in both the TCC and running shoe on the other foot. Data were collected dynamically whilst the participants travelled at approximately 0.4m/sec along a 9m walkway. Both capacitance sensor systems sampled at 50Hz.

## Results

Preliminary analysis reveals that the highest pressure values are visible in two locations on the leg: 1. postero-lateral, along a line posterior to the fibula head and posterior to the lateral malleolus, and; 2. along the lateral border of the tibia. Plantar pressure data indicate that the maximum pressure in the TCC was reduced compared to the running shoe. The healthy participant recorded mean maximum pressure values of 224±28kPa in the running shoe and 159±34kPa in the TCC. This between-shoe trend was similar in the diabetic patient (337±36kPa and 177±18 for the running shoe and cast respectively) but with higher maximum pressures evident in the running shoe.

## Conclusion

This study helps to understand the mechanism of pressure offloading by investigating the interaction between cellular urethane and the ulcer region during walking combined with biomechanical examination of the cast walls and moulding of materials.

